# Oligomeric states of microbial rhodopsins determined by high-speed atomic force microscopy and circular dichroic spectroscopy

**DOI:** 10.1038/s41598-018-26606-y

**Published:** 2018-05-29

**Authors:** Mikihiro Shibata, Keiichi Inoue, Kento Ikeda, Masae Konno, Manish Singh, Chihiro Kataoka, Rei Abe-Yoshizumi, Hideki Kandori, Takayuki Uchihashi

**Affiliations:** 1 Nano Life Science Institute (WPI-NanoLSI), Kanazawa University, Kanazawa, 920-1192 Japan; 2 High-speed AFM for Biological Application Unit, Institute for Frontier Science Initiative, Kanazawa University, Kanazawa, 920-1192 Japan; 3 Department of Life Science and Applied Chemistry, Nagoya Institute of Technology, Nagoya, 464-8555 Japan; 4 OptoBioTechnology Research Center, Nagoya Institute of Technology, Nagoya, 464-8555 Japan; 5 PRESTO, Japan Science and Technology Agency, Kawaguchi, Saitama, 332-0012 Japan; 6 School of Mathematical and Physical Sciences, Graduate School of Natural Science & Technology, Kanazawa University, Kanazawa, 920-1192 Japan; 7 Department of Physics, Nagoya University, Nagoya, 464-8602 Japan; 8 Structural Biology Research Center, Graduate School of Science, Nagoya University, Nagoya, 464-8602 Japan; 9 CREST/JST, 4-1-8 Honcho, Kawaguchi, Saitama 332-0012 Japan

## Abstract

Oligomeric assembly is a common feature of membrane proteins and often relevant to their physiological functions. Determining the stoichiometry and the oligomeric state of membrane proteins in a lipid bilayer is generally challenging because of their large size, complexity, and structural alterations under experimental conditions. Here, we use high-speed atomic force microscopy (HS-AFM) to directly observe the oligomeric states in the lipid membrane of various microbial rhodopsins found within eubacteria to archaea. HS-AFM images show that eubacterial rhodopsins predominantly exist as pentamer forms, while archaeal rhodopsins are trimers in the lipid membrane. In addition, circular dichroism (CD) spectroscopy reveals that pentameric rhodopsins display inverted CD couplets compared to those of trimeric rhodopsins, indicating different types of exciton coupling of the retinal chromophore in each oligomer. The results clearly demonstrate that the stoichiometry of the fundamental oligomer of microbial rhodopsins strongly correlate with the phylogenetic tree, providing a new insight into the relationship between the oligomeric structure and function-structural evolution of microbial rhodopsins.

## Introduction

**Figure 1:**
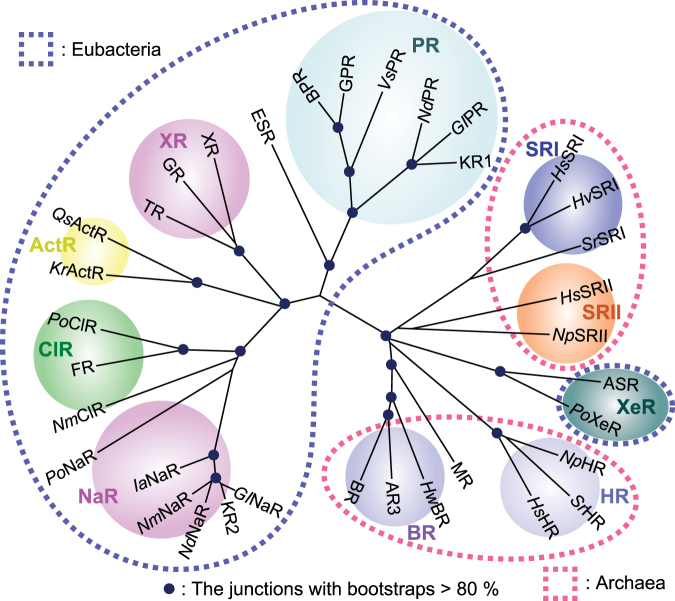
Phylogenetic tree of microbial rhodopsins from eubacteria and archaea. Rhodopsins included in the phylogenetic tree: bacteriorhodopsin from *Halobacterium salinarum* (BR), archaerhodopsin-3 from *Halorubrum sodomense* (AR3), bacteriorhodopsin and middle rhodopsin from *Haloquadratum walsbyi* (*Hw*BR, MR), halorhodopsin from *H. salinarum*, *Salinibacter ruber* and *Natronomonas pharaonis* (*Hs*HR, *Sr*HR, *Np*HR), xenorhodopsin from *Parvularcula oceani* (*Po*XeR), *Anabaena* sensory rhodopsin (ASR), sensory rhodopsin II from *N. pharaonis* and *H. salinarum* (*Np*SRII and *Hs*SRII), sensory rhodopsin I from *S. ruber*, *Haloarcula vallismortis* and *H. salinarum* (*Sr*SRI, *Hv*SRI and *Hs*SRI), proteorhodopsin from *Krokinobacter eikastus*, *Gillisia limnaea*, *Nonlabens dokdonensis* and *Vibrio* sp. AND4 (KR1, *Gl*PR, *Nd*PR, *Vs*PR), blue-absorbing proteorhodopsin from uncultured bacterium (BPR), green-absorbing proteorhodopsin from uncultured marine gamma proteobacterium (GPR), rhodopsin from *Exiguobacterium sibiricum* (ESR), thermophilic rhodopsin from *Thermus thermophilus* (TR), xanthorhodopsin from *S. ruber* (XR), rhodopsin from *Gloeobacter violaceus* PCC 7421 (GR), freshwater actinorhodopsin from *Quadrisphaera* sp. R2A-380-A and *Kineococcus radiotolerans* (*Qs*ActR and *Kr*ActR), eubacterial Cl^−^ pump rhodopsin from *P. oceani*, *Fulvimarina pelagi* and *Nonlabens marinus* S1-08 (*Po*ClR, FR, and *Nm*ClR), putative Na^+^ pump rhodopsin (NaR) from *P. oceani* (*Po*NaR), NaRs from *Indibacter alkaliphilus, N. marinus* S1-08, *N. dokdonensis*, *G. limnaea* (*Ia*NaR, *Nm*NaR, *Nd*NaR, and *Gl*NaR) and KR2.

Microbial rhodopsins (known as type-I rhodopsins) constitute a large group of photoactive membrane proteins with seven-transmembrane α-helices, which are found in archaea, bacteria, and lower eukaryotes (fungi, algae, and some protists) (Fig. 1)^1^. They mainly have an all-*trans* retinal which is photoisomerized to the 13-*cis* form due to photo absorption and exhibit diverse functions such as photosensory signal transduction, active and passive ion transport, and light-regulated enzymatic activity. A recently developed technology, optogenetics, utilizes microbial rhodopsins to control neural activity using light^2,3^. Thus, understanding the fundamental biological properties of microbial rhodopsins is of importance for both basic and application aspects.

Numerous previous studies have revealed various properties relevant to the functional mechanisms of microbial rhodopsins, such as absorption spectra, photocycles, tertiary structures, and light-induced conformational changes^1^. Interestingly, almost all microbial rhodopsins exist as oligomer forms in a native membrane environment. For example, the most well-studied microbial rhodopsin, bacteriorhodopsin (BR), the light-driven proton pump of *Halobacterium salinarum*, forms trimers which pack into a two-dimensional crystal in the native lipid membrane (known as the purple membrane)^4^. The trimeric assembly of BR is considered to be important for its higher thermal stability, while monomeric BR is less stable than the native one^5,6^. The oligomeric assemblies of other microbial rhodopsins are also expected to be relevant for their molecular properties and functionalities. However, although numerous microbial rhodopsins have been recently identified in various organisms, their oligomeric states have remained unclear because of a lack of effective ways to determine them under near a physiological condition^7^.

The oligomeric states of microbial rhodopsins have been mainly studied by X-ray crystallography, gel filtration chromatography, cryo-electron microscopy, and circular dichroism (CD) spectroscopy. However, the determined stoichiometry of the oligomeric structures sometimes led to conflicting results depending on analytical techniques and experimental conditions. For example, crystal structures of sodium pump rhodopsin, *Krokinobacter eikastus* rhodopsin 2 (KR2)^8^, exhibited different oligomer forms depending on the pH conditions: monomers at pH 4.0 and 4.3, and pentamers at pH 4.9 and 5.6^9,10^. X-ray crystallography resolved *Anabaena* sensory rhodopsin (ASR)^11^ as a dimer^12,13^, whereas CD spectroscopy, solid-state nuclear magnetic resonance (SSNMR) spectroscopy and electron paramagnetic resonance (EPR) spectroscopy suggested trimers in lipids^14–16^. The situation for proteorhodopsin (PR) is more complex. The crystal structure of blue-light-absorbing proteorhodopsin (BPR) showed hexamers, while two mutants of HOT75BPR were reported as pentamers^17^. Further, an atomic-force-microscopy (AFM) study of green proteorhodopsin (GPR) showed a coexistence of hexamers and pentamers in the densely packed condition in a lipid membrane^7^. These discordances may originate from different experimental conditions, such as different types of detergents, concentrations of detergents, and pH conditions of crystallization^18^. Thus, conventional measurement techniques are limited for determining the native oligomeric states of microbial rhodopsins.

In this study, we comprehensively analyzed the oligomeric structures of various microbial rhodopsins, including *Krokinobacter eikastus* rhodopsin 2 (KR2: sodium pump), *Gloeobacter* rhodopsin (GR: proton pump), *Fulvimarina* rhodopsin (FR: chloride pump), *Kineococcus radiotolerans* actinorhodopsin (*Kr*ActR: proton pump), *Quadrisphaera* actinorhodopsin (*Qs*ActR: proton pump), GPR (proton pump), *Natronomonas pharaonis* halorhodopsin (*Np*HR: chloride pump), *Natronomonas pharaonis* sensory rhodopsin II (*Np*SRII: phototaxis sensor), *Parvularcula oceani* xenorhodopsin (*Po*XeR: inward proton pump), and *Anabaena* sensory rhodopsin (ASR: photochromic sensor). We applied high-speed atomic force microscopy (HS-AFM) to directly visualize the oligomeric structures of them reconstituted in lipid membranes. HS-AFM is a unique technique that can capture protein dynamics at nanometer resolution under near physiological conditions^19–24^. Previously, we visualized several types of protein dynamics including light-induced conformational changes of BR^25,26^. Although conventional AFM has been successfully applied to observations of microbial rhodopsins such as BR and PR, the slow imaging speed required highly packed conditions in the lipid to suppress the diffusional motion of molecules and enable the high-resolution imaging. In contrast, HS-AFM allows imaging of less packed – and thus staggering – molecules with high resolution^27^.

## Results and Discussion

### Oligomeric structure of sodium-pumping *Krokinobacter* rhodopsin, KR2

**Figure 2:**
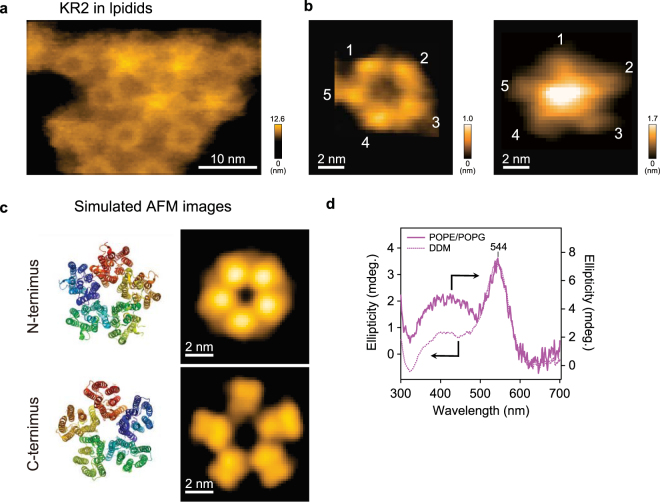
HS-AFM images of KR2. (**a**) Representative HS-AFM image of KR2 incorporated in the lipid. Frame rate, 1 fps. The average number of KR2 oligomers observed in the lipid particles is 15.3 ± 12.3 (mean ± s.d. for 409 pentamers in 28 lipids). (**b**) Magnified views of representative HS-AFM images of KR2 with round (left) and star-like (right) ring arrangements. (**c**) Simulated AFM images of KR2 constructed from the crystal structure of KR2 reported as a pentamer^9^. (**d**) CD spectra of KR2 reconstituted in the lipid (solid line) and solubilized in DDM (dotted line).

First, we analyzed a light-driven sodium-pumping rhodopsin KR2. Crystal structures of KR2 at relatively high (pH 4.0 and 4.3) and low (4.9 and 5.6) acidic pHs were found to contain monomer and pentamer units, respectively^9,10^. HS-AFM images clearly reveal ring-shaped pentameric forms in lipids at physiological pH (pH 8) (Fig. 2a and b). Interestingly, the HS-AFM images exhibit two distinct structures, which could correspond to the two different sides of KR2 in the lipid: C- (cytoplasmic) and N-terminal (extracellular) sides (Fig. 2b). The simulated AFM images of the N- and the C- terminal sides of KR2 constructed from the crystal structure reported as pentamers (PDB code 4XTN)^9^ display two distinctive configurations: a round and a star-like ring shape (Fig. 2c). The simulated AFM image of the N-terminal side reproduces the real AFM image showing the round ring shape well (left panel in Fig. 2b). On the other hand, the simulated AFM image of the C-terminal side is similar to the star-like arrangement of the real AFM image, except for a large protrusion in the ring’s center (right panel in Fig. 2b). This protrusion originates presumably from the additional 6 × His-tag on the C-terminal side of KR2. In fact, after deletion of KR2’s His-tags (KR2_278_), the AFM images show a central pore at the pentamer center similar to the simulated AFM image (see Supplementary Fig. S1a). It is, therefore, safe to conclude that the protrusion seen in Fig. 2b (right panel) corresponds to the additional His-tags of the C-terminal side of KR2. This indicates that the  His-tags on KR2 monomers in the pentamer likely integrate at the center of the pentamer and are rigidly structured. Further, we confirmed that the pentamer form is retained in an acidic solution to compare it with the crystal structure of KR2 prepared at pH 4.0–4.3, which contains the monomeric form^9,10^. All of the KR2_278_ observed at pH 4.3 are pentamers similar to those observed at pH 8.0 (see Supplementary Fig. S1b). This suggests that the monomeric form found in KR2 crystals represents an unphysiological state likely favored during crystallization but is not a native form in the lipid.

**Figure 3:**
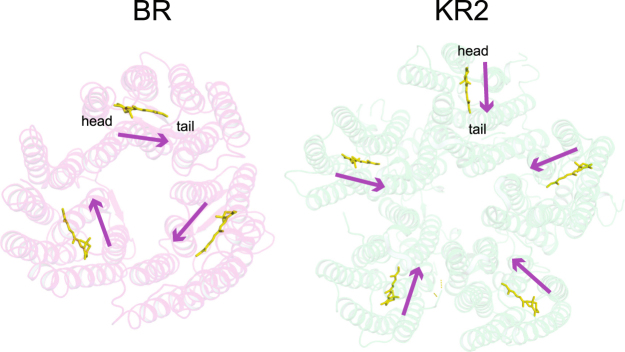
Different orientation of retinals in BR and KR2. The retinals in crystallographic structures of BR trimer (right, PDB ID: 1BRR)^50^ and KR2 pentamer (right, PDB ID: 4XTN)^9^ are highlighted in yellow. Magenta arrows indicate the orientation of retinals.

As described above, visible CD spectroscopy is commonly used to assess the oligomeric structures of microbial rhodopsins. For example, the CD spectra of BR and halorhodopsin from *H. salinarum* (*Hs*HR) show bilobe-shaped spectra (CD couplet) with positive and negative peaks at the short and long wavelength sides of the absorption maximum (λ_max_), respectively^28,29^. The couplet originates from exciton coupling between the retinal chromophores in the trimer^30^. When BR or *Hs*HR is solubilized to the monomeric state, no exciton coupling occurs^29^. Thus, the CD spectrum does not show the bilobed shape and thus only a single positive peak (intrinsic CD) at λ_max_ is observed, whose shape is identical to that of the visible absorption spectrum. In KR2, we observed a single peak around 544 nm, which is similar to those for monomeric BR and *Hs*HR, but the peak wavelength is significantly longer than the λ_max_ (524 nm) (Fig. 2d). This shift to the longer wavelength compared to the λ_max_ can be explained as the sum of intrinsic CD and inverted CD couplets, which have negative and positive peaks at the short and long wavelength sides of λ_max_, respectively. The pattern of exciton coupling strongly depends on the relative orientation of retinal in the oligomer. While the retinals are aligned in a head (the β-ionone ring)-to-tail (the retinal Schiff base) manner in the BR trimer, the  retinals in the KR2 pentamer have a tail-to-tail type orientation (Fig. 3). Thus, the exciton coupling of the retinals in KR2 should differ from that of the retinals in BR, and hence the inverted CD couplet observed in KR2 is expected to be a characteristic of the pentameric oligomer. The CD spectrum of DDM-solubilized KR2 shows an almost identical peak at 544 nm (dotted line in Fig. 2d), indicating that the pentameric form of KR2 is preserved even under DDM-solubilized conditions.

We also attempted to visualize the light-induced conformational changes of KR2 as observed for BR^25^. In BR, our HS-AFM movies clearly showed that the C-terminus of BR monomer undergoes a large conformational change towards the outside of the trimer center^25,26^. Because the photocycle of wild-type KR2 is completed in 8.7 ms, we used an N112A variant of KR2 which has a longer photocycle (1200 ms decay of the M-intermediate) (see Supplementary Fig. S2a)^8,31^. Here, the N112A variant pumps protons, but not sodium ions^8^. The KR2_278_ N112A was observed in the dark and after green light illumination (see Supplementary Fig. S2b and Video S1). Unlike BR, clear conformational changes of KR2 triggered by light were not detected, although we imaged both the C- and N-terminal sides. This result suggests that KR2 does not undergo large conformational changes which can be resolved by our HS-AFM during the photocycle. Translocation of sodium ions inside KR2 may occur by diffusion without large conformational changes.

### Oligomeric structures of microbial rhodopsins from eubacteria

**Figure 4:**
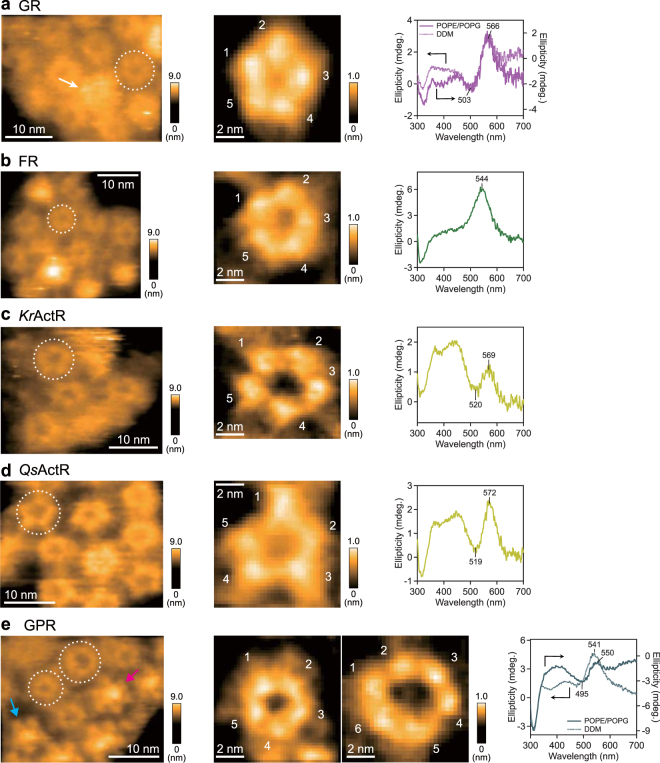
Oligomeric states of microbial rhodopsins from eubacteria. (left panels) Typical HS-AFM images of (**a**) GR, (**b**) FR, (**c**) *Kr*ActR, (**d**) *Qs*ActR and (**e**) GPR, reconstituted in the lipids. Frame rate, 1 fps. The average numbers of oligomers observed in the lipid particles were (GR) 45.4 ± 29.1 (mean ± s.d. for 409 pentamers in 9 lipids), (FR) 46.0 ± 19.3 (mean ± s.d. for 414 pentamers in 9 lipids), (*Kr*ActR) 22.6 ± 14.6 (mean ± s.d. for 317 pentamers in 14 lipids), (*Qs*ActR) 25.0 ± 18.2 (mean ± s.d. for 150 pentamers in 6 lipids), (GPR) 32.6 ± 14.0 (mean ± s.d. for 424 pentamers/hexamers in 13 lipids). Broken circles on the images indicate round-ring pentamers. A white arrow in the left panel of (**a**) indicates the star-like arrangement of the GR pentamer. Cyan and magenta arrows in (**e**) indicate the pentamer and the hexamer of GPR, respectively. (middle panels) Magnified views of round-ring oligomers for each eubacterial rhodopsin are shown with the number of subunits (1–6). (right panels) CD spectra of (**a**) GR, (**b**) FR, (**c**) *Kr*ActR, (**d**) *Qs*ActR and (**e**) GPR reconstituted in the lipids (solid lines) and solubilized in DDM (dotted lines).

Next, we observed other microbial rhodopsins, recently identified in different species, to systematically determine their oligomer states. Figure 4a shows an AFM image of proton-pumping GR in the lipid, revealing that GR is in a pentameric form similar to KR2 (left and middle panels of Fig. 4a). In addition, the GR pentamers also exhibit both round ring and star-like arrangements similar to the KR2 pentamers (left panel of Fig. 4a). However, the protrusion at the center of the pentamer is more blurred compared to that of KR2, although the GR monomers also have a 6 × His-tag at the C-terminal side. This may represent structural differences in the C-terminal sides between KR2 and GR monomers.

The CD spectrum of GR in the lipid (POPE/POPG) shows a couplet with a negative peak at 503 nm and positive peak at 566 nm (right panel of Fig. 4a), which is clearer than that of KR2. This blue-side negative/red-side positive couplet suggests that GR also forms pentamers like KR2, which is consistent with the AFM image. The CD spectrum of solubilized GR in DDM shows a similar CD couplet to that in the lipid, indicating that the solubilization does not change the oligomer form of GR. A previous report showing the similar CD couplets both in detergent and an *E. coli* membrane concluded that the inverted CD couplet of GR represents the formation of trimers with different molecular arrangements from BR and *Np*HR^32^. However, the combination of HS-AFM and CD spectroscopy indicates that the inverted CD spectrum is a characteristic of pentamer form. In fact, the size-exclusion chromatography pattern in the previous report showed that the main peak of GR in DDM was significantly higher than that of *Np*HR^32^, which was considered to be in the trimeric form^33–35^. This also suggests that GR forms larger oligomers than trimers even in DDM.

In the same way, we analyzed the oligomeric state of inward chloride-pumping FR. The AFM image of FR clearly indicates the pentameric form in the lipid (left and middle panels of Fig. 4b). The CD spectrum of FR shows a single positive peak at 544 nm whose the wavelength is longer than the λ_max_ of FR (518 nm)^36^ and is similar to that of KR2 (right panel of Fig. 4b). Furthermore, we evaluated the oligomeric states of *Kr*ActR and *Qs*ActR belonging to the recently reported actinorhodopsin sub-family of proton pumps found in freshwater (Fig. 1)^37^. Both the HS-AFM images of *Kr*ActR and *Qs*ActR show the ring-like pentamer forms (left and middle panels of Fig. 4c and d), same as KR2, GR, and FR. The CD spectra of *Kr*ActR and *Qs*ActR show positive peaks at 569 and 572 nm, which are longer than the λ_max_ (*Kr*ActR: 532 nm; *Qs*ActR: 551 nm), respectively (right panels in Fig. 4c and d). These results suggest that *Kr*ActR and *Qs*ActR also have negative contribution of the couplet at the blue side of their absorption peaks. They are observed as dips at 520 and 519 nm, respectively, and do not decrease below zero because of the overlapping positive intrinsic CD. Thus, the orientation of retinal chromophores in ActR pentamers is identical to those of KR2, GR, and FR. Interestingly, the AFM image of ActR does not show protrusions at the center of the pentameric ring, in contrast to the KR2, GR, and FR. This is likely because the ring diameters of ActR pentamers (4.5 ± 0.21 nm for *Kr*ActR and 4.9 ± 0.050 nm for *Qs*ActR) are larger than those of the others (4.3 ± 0.070 nm for KR2, 4.3 ± 0.12 nm for GR and 4.1 ± 0.26 nm for FR), and therefore entanglement of the His-tags would hardly occur. This also implies a looser interaction between monomers in the ActR compared to in KR2, GR, and FR. Furthermore, both *Kr*ActR and *Qs*ActR have longer C-terminal tails than KR2 and FR after the end of 7th transmembrane helix (helix G) (see Supplementary Table 1). The longer tails might have large fluctuations and inhibit the formation of the rigid protrusion structure. Similarly, GR has a longer C-terminal tail than KR2. This can also facilitate large fluctuations and a less-structured protrusion.

A previous AFM study showed that proton-pumping GPR predominantly assembles into a hexameric form with a smaller fraction assembling into a pentameric form in the densely packed condition in a lipid membrane^7^. Our AFM images of GPR in a lipid membrane also reveal the coexistence of pentamers and hexamers (Fig. 4e), although pentamers are the dominant structure in our experimental conditions (pentamers: hexamers = 76% : 24% for 486 oligomers in total). The CD spectrum of GPR shows a couplet with a negative peak at 495 nm and positive peak at 550 nm in the lipid (rightmost panel in Fig. 4e). This negative/positive couplet is similar to those of other eubacterial rhodopsins (KR2, FR, *Kr*ActR and *Qs*ActR), and consistent with the pentamer dominant oligomeric state of GPR observed by HS-AFM. The inhomogeneous oligomeric mixture of GPR suggests that inter-molecular interaction is weaker than that of other eubacterial rhodopsins which form a strictly regulated pentameric structure. Since this indicates a decreased robustness of GPR oligomers, the CD spectrum was also measured in DDM. For the solubilized GPR, we observed a large change in the CD spectrum with a single positive peak at 541 nm. This is still significantly longer than the λ_max_ of GPR (519 nm), indicating that GPR forms pentamers even in the detergent. The less significant contribution of the couplet, however, compared with that in the lipid implies that a part of the oligomeric mixture of pentamers and hexamers is disrupted and/or the exciton coupling is weakened by the change in the oligomeric structure in the solubilized condition. Taken together, the native oligomeric states of most eubacterial rhodopsins appear to be mainly pentamers. Interestingly, however, these microbial rhodopsins transport different ions in opposite directions, suggesting that oligomer formation is not crucial for the fundamental functions of microbial rhodopsins.

### Oligomeric structures of XeR and microbial rhodopsins from archaea

**Figure 5:**
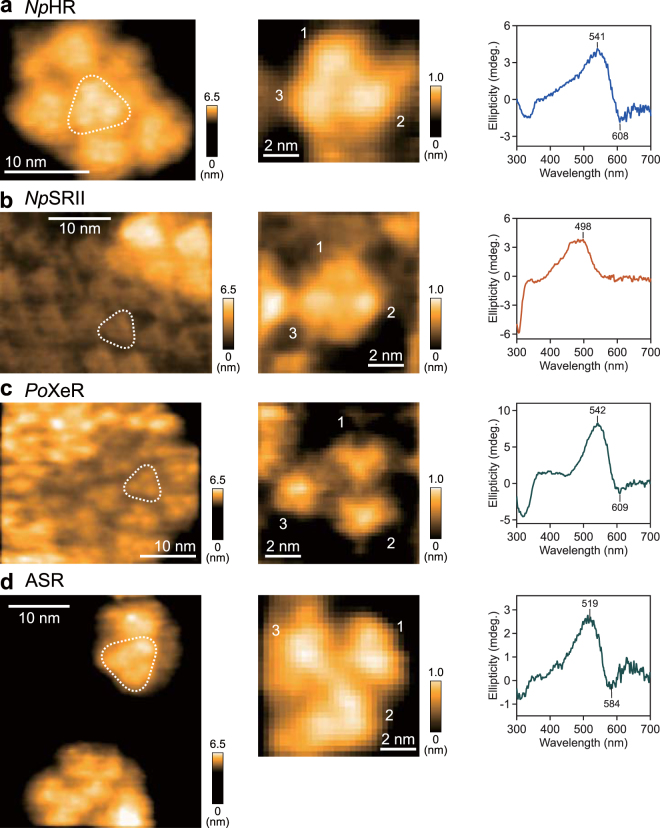
Oligomeric states of XeR and microbial rhodopsins from archaea. (left panels) Typical HS-AFM images of (**a**) *Np*HR, (**b**) *Np*SRII, (**c**) *Po*XeR and (**d**) ASR reconstituted in the lipids. Frame rate, 1 fps. The average numbers of oligomers observed in the lipid particles were (*Np*HR) 9.1 ± 7.8 (mean ± s.d. for 201 trimers in 22 lipids), (*Np*SRII) 45.5 ± 31.3 (mean ± s.d. for 273 trimers in 6 lipids), (*Po*XeR) 50.0 ± 20.4 (mean ± s.d. for 150 trimers in 3 lipids), (ASR) 4.1 ± 1.7 (mean ± s.d. for 115 trimers in 28 lipids). Dotted triangles on the images indicate the trimer units. (middle panels) Magnified views of trimers for each microbial rhodopsin are shown with the number of subunits (1–3). (right panels) CD spectra of reconstituted (**a**) *Np*HR, (**b**) *Np*SRII, (**c**) *Po*XeR and (**d**) ASR in the lipids.

It is well-known that the fundamental oligomeric state of BR from archaea is a trimer in the native purple membrane^4^. Further, trimeric oligomers of some microbial rhodopsins from archaea have been determined using various methods^34^, but there has been no direct evidence under lipid-reconstituted conditions. The AFM image of chloride pump *Np*HR clearly shows the trimer forms in the lipid, which is consistent with a previous report (Fig. 5a)^34^. Additionally, the CD spectrum of *Np*HR shows a positive peak and negative peak on the blue (541 nm) and red sides (608 nm) of λ_max_ = 576 nm, respectively (right panel in Fig. 5a)^38^. This is similar to the CD spectra of trimeric BR and *Hs*HR^28,29^, and the CD couplet is inverted compared to those observed in the pentameric rhodopsins (KR2, FR, GR, *Kr*ActR, *Qs*ActR and GPR). Thus, *Np*HR forms trimer as observed with AFM in the lipid membrane, which is also suggested by crystallography^35^. The blue-side-positive/red-side-negative type of CD couplet would be a common signature of trimeric rhodopsins.

Next, we observed archaeal phototaxis receptor sensory rhodopsin II from *N. pharaonis* (*Np*SRII) incorporated into the lipid. *Np*SRII was found to form trimers, by AFM (Fig. 5b). The CD spectrum of *Np*SRII shows only a positive peak at 498 nm, which is nearly identical to the λ_max_ (500 nm)^39^ (right panel in Fig. 5b). This indicates that the CD couplet by exciton coupling between *Np*SRII molecules is much smaller than the intrinsic CD, although NpSRII molecules are in the trimeric form. Consequently, the oligomers of microbial rhodopsins from archaea typically predominantly exist as trimers in the lipid.

The xenorhodopsin (XeR) group is found in eubacteria, but is classified as an archaeal type rhodopsin in the phylogenetic tree of microbial rhodopsins (Fig. 1). We recently reported that inward proton-pumping xenorhodopsin from *Parvularcula oceani* (*Po*XeR) forms trimers in a lipid^40^. We reproduced the previous result that *Po*XeR trimers pack into a 2D crystal similar to BR (left panel of Fig. 5c). The CD couplet of *Po*XeR shows positive and negative peaks at 542 and 609 nm, which are shorter and longer than the λ_max_ (569 nm), respectively^40^, confirming that *Po*XeR exists in the trimeric form in the lipid (right panel of Fig. 5c). Furthermore, we observed ASR which belongs to the same group as *Po*XeR (Fig. 1). X-ray crystal analysis of ASR suggested a dimeric oligomer^12,13^, while CD spectroscopy, SSNMR spectroscopy, and EPR spectroscopy suggested trimers in lipids^15^. In addition, a double electron-electron resonance EPR study using site-directed spin labeling of two ASR cysteine mutants confirmed the trimeric nature of ASR in lipids^16^. Also, both AFM images and CD spectrum indicate trimeric form for ASR (right panel of Fig. 5d), which is in good agreement with other experimental results. The dimeric form resolved by crystallography could be caused by crystallization processes of the solubilized molecules. Taken together, XeR likely exists as a trimer, similarly to BR, *Np*HR and *Np*SRII.

In Fig. 5a,b and c, the trimers in the two-dimensional packing show two different heights. It has been reported that the cytoplasmic side of BR is more protruded from the membrane surface than the extracellular side^41^. Thus, the height (image contrast) differences of trimers are probably caused by the height differences between the cytoplasmic (C-terminal) and the extracellular (N-terminal) sides of molecules protruded from the membrane surface.

Among four types of trimeric rhodopsins (*Np*HR, *Np*SRII, *Po*XeR, and ASR), only the CD couplet of *Np*SRII is very small. This may indicate different packing of *Np*SRII in the trimer compared to the others. The outer diameter of *Np*SRII trimers (2.8 ± 0.11 nm) is smaller than those of the others (3.5 ± 0.27 nm for *Np*HR, 4.0 ± 0.33 nm for *Po*XeR and 4.8 ± 0.36 nm for ASR). This suggests that *Np*SRII forms trimeric structures that are tighter packed compared to BR or ASR which have stronger CD couplets. Therefore, the relative orientation of retinals in *Np*SRII is different from those of BR and ASR, resulting in a loss of the CD couplet in the spectrum.

### Correlation between stoichiometry of oligomers and the phylogenetic tree of microbial rhodopsins

**Figure 6:**
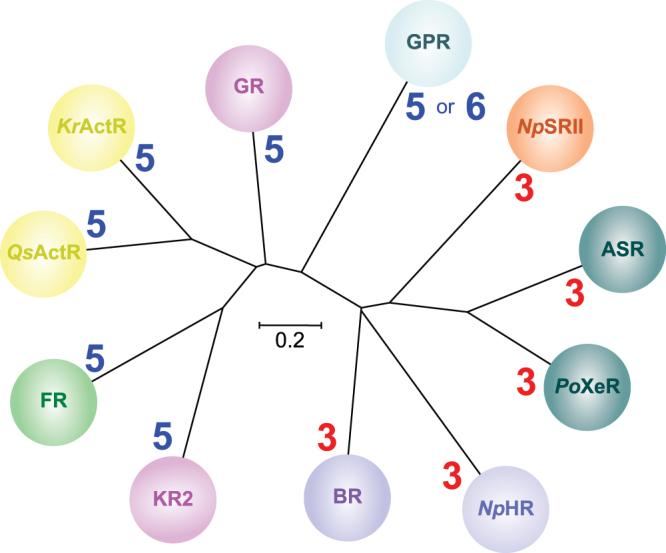
Phylogenetic tree of microbial rhodopsins with stoichiometry of oligomeric states determined by HS-AFM and CD spectroscopy.

We systematically observed various microbial rhodopsins reconstituted into a lipid membrane by HS-AFM and CD spectroscopy, and determined their native oligomeric states. Based on the HS-AFM and CD spectroscopic analyses, the fundamental oligomeric states of microbial rhodopsins can be classified into two groups; one is a trimeric form and the other is a pentameric form. Taking the phylogenetic tree of microbial rhodopsins into consideration, the branching point of the trimer and pentamer likely emerged at an early stage of evolution (Fig. 6). Whereas all pentameric rhodopsins (KR2, GR, FR, *Kr*ActR, *Qs*ActR, and GPR) originate from eubacteria, archaeal rhodopsins (BR, *Np*HR, and *Np*SRII) form trimers. Thus, pentameric and trimeric rhodopsins likely evolved after branching of archaea from eubacteria. The only exception from this rule is XeRs (ASR and *Po*XeR). ASR and *Po*XeR form trimers, but are present in the eubacteria, *Anabaena* sp. PCC7120 and *P. oceani*, respectively. Although further studies are needed to determine the evolutionary relationship between XeRs and other archaeal rhodopsins, many eubacteria contain genes derived from archaeal species by lateral gene transfer^42^. Thus, the XeR group likely evolved from some eubacteria after lateral transfer of genes of trimeric rhodopsins from archaeal species.

Whole microbial rhodopsins have a seven-transmembrane structure, but the oligomeric states are distinctly different. The factors affecting these different oligomeric structures remain elusive. To address this issue, we compared the amino acid sequences of microbial rhodopsins used in this study (see Supplementary Table 1). However, there are no completely conserved amino acid residues at the protein interfaces, nor specificity among trimers and pentamers. These results strongly suggest that a single specific amino acid does not play a crucial role in oligomer formation, and integration of several non-polar interactions and shapes at the protein interface is likely essential for determining the oligomeric state.

Eukaryotic microbial rhodopsins such as channel rhodopsins exist in a dimeric form, stabilized by a disulfide bond between molecules^43,44^. This indicates that the stability and function of eukaryotic rhodopsin are optimized by dimer formation and that the molecular interactions differ from those in eubacterial and archaeal proteins. The functional units of many channels are tetramers and form pores to transport ions. Additionally, some receptors, such as glutamate receptors (NMDAR and AMPAR) form tetramers, while visual rhodopsin, G protein-coupled receptors, and receptor tyrosine kinases form dimers in lipids. Further experimental studies are required to address these aspects; if an ancestor of microbial rhodopsin is identified, the biological meaning of trimer and pentamer formation in the evolutionary process can be determined.

## Materials and Methods

### Sample preparation

We used synthesized and *E. coli* codon-optimized genes for protein expression of KR2, FR, *Kr*ActR, *Qs*ActR, and *Po*XeR. All genes were inserted into the multi-cloning site of the pET21a vector (Novagen, Madison, WI, USA) between the *Nde*I and *Xho*I restriction sites^40^. The genetic constructs used to express GR, GPR, *Np*HR, *Np*SRII and ASR have been described previously^38,39,45–47^. Microbial rhodopsins were expressed in *E. coli* cells in the following strains: *Np*HR and *Np*SRII: BL21(DE3); ASR, KR2 (wt, KR2_278_ and N112A), FR and GR: C41(DE3); GPR, *Kr*ActR and *Qs*ActR: C43(DE3). All proteins were solubilized in 1.5% *n*-dodecyl-β-D-maltoside (DDM) (Anatrace, OH, USA) and then purified by Co-affinity column (TALON, Qiagen, Hilden, Germany) chromatography^40^. The final protein concentration was adjusted to 2.5 mg/mL for the reconstitution into the lipid membrane.

KR2_278_ was constructed in pET21a vector with 6 residues deleted at the C-terminus of the KR2 gene, and contained a thrombin cleavage site and 6 × His tag at the 3′ end. The thrombin cleavage site and 6 × His tag was attached at the C-terminus of KR2-derived genes by polymerase chain reaction using PrimeSTAR Max DNA Polymerase (Takara Bio, Shiga, Japan). *Nde*I and *Xho*I restriction sites were introduced at the 5′ and 3′ ends, respectively. The gene was then inserted into the pET21a vector. The expression vectors were transformed into the *E. coli* C43 (DE3) strain. After protein purification, the 6 × His tag was removed by overnight treatment with 10 U/mg protein of thrombin at 4 °C. To remove uncleaved His-tag molecules, thrombin-treated protein was purified using Micro-spin columns (Thermo Scientific, Waltham, MA, USA) containing Co-NTA resin. The flow-through fraction was used in each experiment.

### Preparation of lipid-reconstituted microbial rhodopsins

In general, reconstituted proteoliposomes have been used to incorporate membrane proteins into lipids. For AFM imaging, the proteoliposome is ruptured on a solid substrate and then the supported lipid membrane containing proteins is imaged. However, HS-AFM observations of the ruptured proteoliposomes showed large bending of the lipid under dense conditions (see Supplementary Fig. S3a). At dilute protein concentrations, images of proteins on the flat membrane area were unclear, likely because of the rapid diffusion of molecules. Instead, we observed aggregates at the edge of the membrane (see Supplementary Fig. S3b). Membrane bending and aggregation prevented high-resolution imaging by HS-AFM. To overcome these problems, we applied membrane scaffolding proteins (MSP), which were developed for nanodisc technology^48,49^. Because of the limited size of the membranes used with MSP, diffusion of membrane proteins in the supported membrane can be suppressed. Additionally, the membrane can be maintained as a flat sheet by MSP. Therefore, microbial rhodopsins reconstituted into the size-limited membrane can be used to obtain high-quality AFM images, which are crucial for determining the oligomeric states of microbial rhodopsins.

We followed the manufacturer’s protocol for the nanodisc (Sigma-Aldrich, St. Louis, MO, USA) with minor modifications. Briefly, for reconstituted lipids, we used a mixture of phospholipids, asolectin from soybean (Sigma-Aldrich, No. 11145). Asolectin (120 μg) was dissolved in chloroform and then evaporated under N_2_ gas to completely remove the solvent. Next, the lipids were suspended in 50 μL buffer A (20 mM HEPES-KOH (pH 7.4), 100 mM NaCl_2_, and 4% DDM) and sonicated for ~1 min with a tip sonicator. Next, dissolved membrane proteins (1 nmol) and MSP (50 μL, 1 mg/mL) (MSP1E3D1, Sigma-Aldrich, No. M7074) were added to the lipid suspension and mixed for ~1 h while rotating in the dark at 4 °C. Finally, we added 60 mg Bio-beads SM-2 (Bio-Rad, Hercules, CA, USA, No. 1523920) and dialyzed the samples in detergent overnight at 4 °C. According to the manufacturer’s protocol, nanodisc samples should be fractionated on a column to purify the nanodiscs based on size (~10 nm in diameter). Here, we did not purify the reconstituted samples, but obtained flat membranes with limited sizes <30 nm in diameter.

### High-speed AFM measurements

A laboratory-built HS-AFM operated in tapping mode was used^19,26^. Briefly, cantilever deflection was detected with an optical beam deflection detector using an infrared laser (0.7 mW, 780 nm). The infrared laser beam was focused onto the back side of a cantilever (Olympus, Tokyo, Japan: BL-AC7DS-KU4) covered with a gold film through a ×60 objective lens (Nikon, Tokyo, Japan: CFI S Plan Fluor ELWD 60×) and the reflected infrared laser was detected by a two-segmented PIN photodiode. The cantilever was 6–7 μm long, 2 μm wide, and 90 nm thick. The spring constant, resonant frequency, and quality factor were 0.1–0.2 N/ m, ∼1 MHz, and ∼2 in liquid, respectively. The free oscillation amplitude was ∼1 nm and set-point amplitude was approximately 90% of the free amplitude for the feedback control of HS-AFM observation. An amorphous carbon tip, grown by electron beam deposition, was used as an AFM probe. The length of the electron beam deposition tip was ∼500 nm, and the end radius of the tip was ∼4 nm. For the photo-illumination experiment of KR2, a green laser with a wavelength of 532 nm was periodically irradiated onto the sample through the objective lens during the imaging. As a substrate, a mica surface treated with 0.01% (3-aminopropyl)triethoxysilane (Shin-Etsu Silicone, Tokyo, Japan) was used. HS-AFM experiments were carried out in a buffer solution containing 20 mM Tris–HCl (pH 8.0) and 50 mM NaCl, or 20 mM citrate-NaOH (pH 4.3), 50 mM NaCl at room temperature.

### Circular dichroic spectroscopy measurements

For CD spectroscopy, rhodopsins were reconstituted into liposomes composed of a mixture of 1-palmitoyl-2-oleoyl-sn-glycero-3-phosphoethanolamine (POPE) (Avanti Polar Lipids, Alabaster, AL, USA) and 1-palmitoyl-2-oleoyl-sn-glycero-3-phospho-(1′-rac-glycerol) (POPG) (Avanti Polar Lipids) (molar ratio = 3:1) in 100 mM NaCl and 20 mM Tris-HCl (pH 8.0) as previously reported^40^. Upon reconstitution, the molar ratio between protein and lipids was adjusted to 1:50. The concentration of proteoliposome was adjusted to have an absorption of 0.8–0.9 for the visible absorption peak of retinal. CD spectra were obtained using a CD spectrometer (J-1500, JASCO, Tokyo, Japan). The spectra were scanned in the region of 300–750 nm 8–24 times to obtain higher signal-to-noise ratios at a scan rate of 200 nm/min at 25 °C.

### Construction of simulated AFM image

To compare the HS-AFM images of KR2 with its previously reported crystal structure^9^, we simulated AFM images using a simple hard sphere model. The AFM probe was modeled as a cone shape with a half angle of 5° and an end radius of 0.5 nm. Simulation was carried out for either the N- or C terminus side of the crystal structure of KR2 (PDB code, 4XTN). The simulated images were processed with a low-pass filter with a cut-off wavelength of 2 nm because the spatial resolution of the AFM image is typically limited to approximately 2 nm.

### Data availability

The raw AFM image files and the raw data of CD spectra are available from the authors on request.

## Electronic supplementary material

Supplementary information

The HS-AFM movie of KR2278 N112A under periodical light irradiation.

## References

[1] Ernst OP (2014). Microbial and animal rhodopsins: structures, functions, and molecular mechanisms. Chem Rev.

[2] Deisseroth K (2015). Optogenetics: 10 years of microbial opsins in neuroscience. Nat Neurosci.

[3] Zhang F (2011). The microbial opsin family of optogenetic tools. Cell.

[4] Henderson R, Unwin PN (1975). Three-dimensional model of purple membrane obtained by electron microscopy. Nature.

[5] Brouillette CG, McMichens RB, Stern LJ, Khorana HG (1989). Structure and thermal stability of monomeric bacteriorhodopsin in mixed phospholipid/detergent micelles. Proteins.

[6] Heyes CD, El-Sayed MA (2002). The role of the native lipids and lattice structure in bacteriorhodopsin protein conformation and stability as studied by temperature-dependent Fourier transform-infrared spectroscopy. J Biol Chem.

[7] Klyszejko AL (2008). Folding and assembly of proteorhodopsin. J Mol Biol.

[8] Inoue K (2013). A light-driven sodium ion pump in marine bacteria. Nat Commun.

[9] Gushchin I (2015). Crystal structure of a light-driven sodium pump. Nat Struct Mol Biol.

[10] Kato HE (2015). Structural basis for Na^+^ transport mechanism by a light-driven Na^+^ pump. Nature.

[11] Jung KH, Trivedi VD, Spudich JL (2003). Demonstration of a sensory rhodopsin in eubacteria. Mol Microbiol.

[12] Vogeley L (2004). Anabaena sensory rhodopsin: a photochromic color sensor at 2.0 Å. Science.

[13] Dong B, Sanchez-Magraner L, Luecke H (2016). Structure of an Inward Proton-Transporting *Anabaena* Sensory Rhodopsin Mutant: Mechanistic Insights. Biophys J.

[14] Wang S (2012). Paramagnetic relaxation enhancement reveals oligomerization interface of a membrane protein. J Am Chem Soc.

[15] Ward ME (2015). *In situ* structural studies of *Anabaena* sensory rhodopsin in the *E. coli* membrane. Biophys J.

[16] Milikisiyants S (2017). Oligomeric Structure of *Anabaena* Sensory Rhodopsin in a Lipid Bilayer Environment by Combining Solid-State NMR and Long-range DEER Constraints. J Mol Biol.

[17] Ran T (2013). Cross-protomer interaction with the photoactive site in oligomeric proteorhodopsin complexes. Acta Crystallogr D Biol Crystallogr.

[18] Brown LS, Ernst OP (2017). Recent advances in biophysical studies of rhodopsins - Oligomerization, folding, and structure. Biochim Biophys Acta.

[19] Ando T (2001). A high-speed atomic force microscope for studying biological macromolecules. Proc Natl Acad Sci USA.

[20] Kodera N, Yamamoto D, Ishikawa R, Ando T (2010). Video imaging of walking myosin V by high-speed atomic force microscopy. Nature.

[21] Igarashi K (2011). Traffic jams reduce hydrolytic efficiency of cellulase on cellulose surface. Science.

[22] Uchihashi T, Iino R, Ando T, Noji H (2011). High-speed atomic force microscopy reveals rotary catalysis of rotorless F_1_-ATPase. Science.

[23] Ando T, Uchihashi T, Scheuring S (2014). Filming biomolecular processes by high-speed atomic force microscopy. Chem Rev.

[24] Shibata M (2017). Real-space and real-time dynamics of CRISPR-Cas9 visualized by high-speed atomic force microscopy. Nat Commun.

[25] Shibata M, Yamashita H, Uchihashi T, Kandori H, Ando T (2010). High-speed atomic force microscopy shows dynamic molecular processes in photoactivated bacteriorhodopsin. Nat Nanotechnol.

[26] Shibata M, Uchihashi T, Yamashita H, Kandori H, Ando T (2011). Structural changes in bacteriorhodopsin in response to alternate illumination observed by high-speed atomic force microscopy. Angew Chem Int Ed Engl.

[27] Yamashita H (2013). Role of trimer-trimer interaction of bacteriorhodopsin studied by optical spectroscopy and high-speed atomic force microscopy. J Struct Biol.

[28] Pescitelli G, Woody RW (2012). The exciton origin of the visible circular dichroism spectrum of bacteriorhodopsin. J Phys Chem B.

[29] Hasselbacher CA, Spudich JL, Dewey TG (1988). Circular dichroism of halorhodopsin: comparison with bacteriorhodopsin and sensory rhodopsin I. Biochemistry.

[30] Fujimoto KJ (2010). Transition-density-fragment interaction approach for exciton-coupled circular dichroism spectra. J Chem Phys.

[31] Inoue K, Konno M, Abe-Yoshizumi R, Kandori H (2015). The Role of the NDQ Motif in Sodium-Pumping Rhodopsins. Angew Chem Int Ed Engl.

[32] Tsukamoto T (2013). Salt bridge in the conserved His-Asp cluster in *Gloeobacter* rhodopsin contributes to trimer formation. FEBS Lett.

[33] Havelka WA, Henderson R, Oesterhelt D (1995). Three-dimensional structure of halorhodopsin at 7 A resolution. J Mol Biol.

[34] Sasaki T (2009). Halorhodopsin from *Natronomonas* pharaonis forms a trimer even in the presence of a detergent, dodecyl-beta-D-maltoside. Photochem Photobiol.

[35] Kouyama T (2010). Crystal structure of the light-driven chloride pump halorhodopsin from *Natronomonas* pharaonis. J Mol Biol.

[36] Inoue K, Koua FH, Kato Y, Abe-Yoshizumi R, Kandori H (2014). Spectroscopic study of a light-driven chloride ion pump from marine bacteria. J Phys Chem B.

[37] Sharma AK, Zhaxybayeva O, Papke RT, Doolittle WF (2008). Actinorhodopsins: proteorhodopsin-like gene sequences found predominantly in non-marine environments. Environ Microbiol.

[38] Muroda K, Nakashima K, Shibata M, Demura M, Kandori H (2012). Protein-bound water as the determinant of asymmetric functional conversion between light-driven proton and chloride pumps. Biochemistry.

[39] Nakatsuma A (2011). Chimeric microbial rhodopsins containing the third cytoplasmic loop of bovine rhodopsin. Biophys J.

[40] Inoue K (2016). A natural light-driven inward proton pump. Nat Commun.

[41] Müller DJ, Schoenenberger CA, Büldt G, Engel A (1996). Immuno-atomic force microscopy of purple membrane. Biophys J.

[42] Mongodin EF (2005). The genome of Salinibacter ruber: convergence and gene exchange among hyperhalophilic bacteria and archaea. Proc Natl Acad Sci USA.

[43] Pescitelli G (2014). Exciton circular dichroism in channelrhodopsin. J Phys Chem B.

[44] Kato HE (2012). Crystal structure of the channelrhodopsin light-gated cation channel. Nature.

[45] Sasaki K (2014). Chimeric proton-pumping rhodopsins containing the cytoplasmic loop of bovine rhodopsin. PLoS One.

[46] Kawanabe A, Furutani Y, Jung KH, Kandori H (2007). Photochromism of *Anabaena* sensory rhodopsin. J Am Chem Soc.

[47] Ozaki Y, Kawashima T, Abe-Yoshizumi R, Kandori H (2014). A color-determining amino acid residue of proteorhodopsin. Biochemistry.

[48] Bayburt TH, Grinkova YV, Sligar SG (2002). Self-assembly of discoidal phospholipid bilayer nanoparticles with membrane scaffold proteins. Nano Lett.

[49] Denisov IG, Sligar SG (2016). Nanodiscs for structural and functional studies of membrane proteins. Nat Struct Mol Biol.

[50] Essen L, Siegert R, Lehmann WD, Oesterhelt D (1998). Lipid patches in membrane protein oligomers: crystal structure of the bacteriorhodopsin-lipid complex. Proc Natl Acad Sci USA.

