# Supplementary material for: Oligomeric states of microbial rhodopsins determined by high-speed atomic force microscopy and circular dichroic spectroscopy
Source: Sci Rep. 2018 May 29;8:8262. doi: 10.1038/s41598-018-26606-y (PMC5974397; doi:10.1038/s41598-018-26606-y)
Supplement: Supplementary file 1 — Supplementary information [file 41598_2018_26606_MOESM1_ESM.docx]

**Supplementary information**

**Oligomeric states of microbial rhodopsins determined by high-speed atomic force microscopy and circular dichroic spectroscopy**

Mikihiro Shibata^+^, Keiichi Inoue^+^, Kento Ikeda, Masae Konno, Manish Singh, Chihiro Kataoka, Rei Abe-Yoshizumi, Hideki Kandori^*^, and Takayuki Uchihashi^*^

^+^these authors contributed equally to this work

*Correspondence and requests for materials should be addressed to H. K. (kandori@nitech.ac.jp) and T. U. (uchihast@d.phys.nagoya-u.ac.jp)

This PDF includes:

Supplementary Table S1

Supplementary Figures S1-S3

Legend to Supplementary Video S1

**
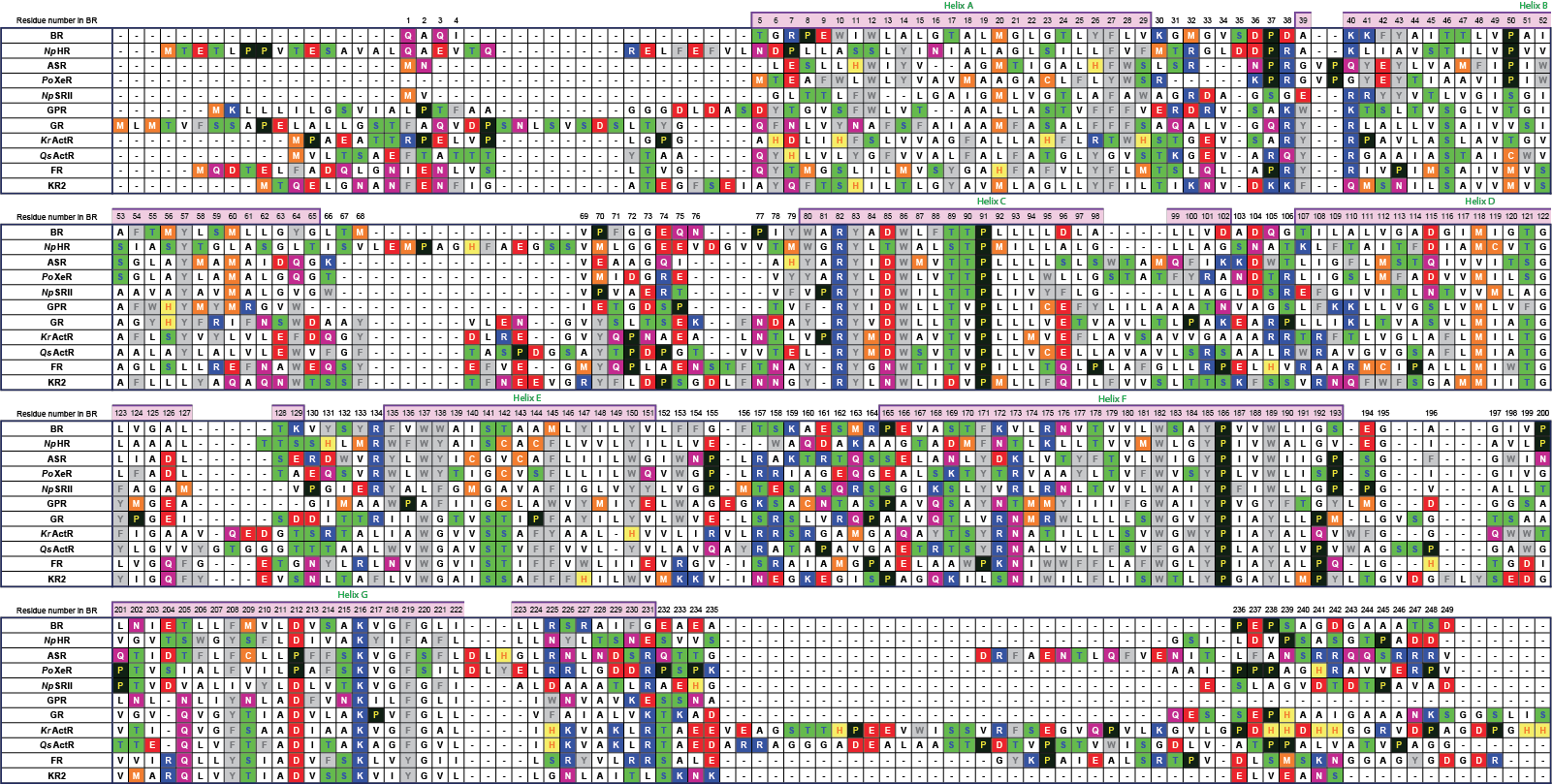
**

**Supplementary Table S1 | Alignment of amino acid residues in microbial rhodopsins.** Alignment of amino acid sequences of BR, *Np*HR, ASR, *Po*XeR, *Np*SRII, GPR, GR, *Kr*ActR, *Qs*ActR, FR, and KR2. Residue numbers in BR sequence are shown on the top. The positions of transmembrane helices are indicated by purple rectangles for BR according to the X-ray crystallographic structure (PDB ID: 1BRR).

**
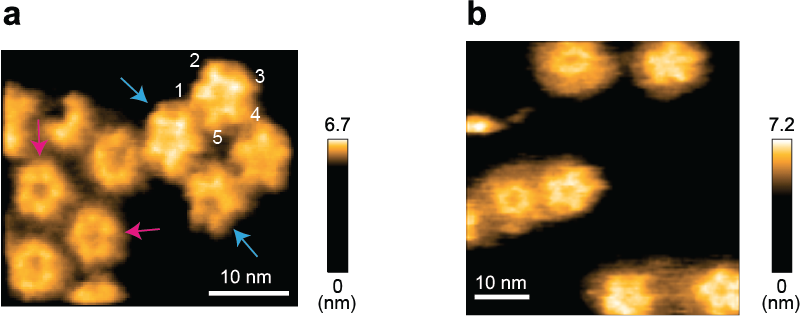
**

**Supplementary Figure S1 | AFM images of KR2 deleted His-tag (KR2_278_) at different pH conditions.** (**a**) Representative HS-AFM image of KR2_278_ in the lipids observed at pH 8.0. Magenta and cyan arrows indicate the N-terminal (extracellular) and the C-terminal (cytoplasmic) faces of the pentamers, respectively. Frame rate, 1 fps. (**b**) Representative HS-AFM image of KR2_278_ in the lipids observed at pH 4.3. Frame rate, 3.3 fps.

**
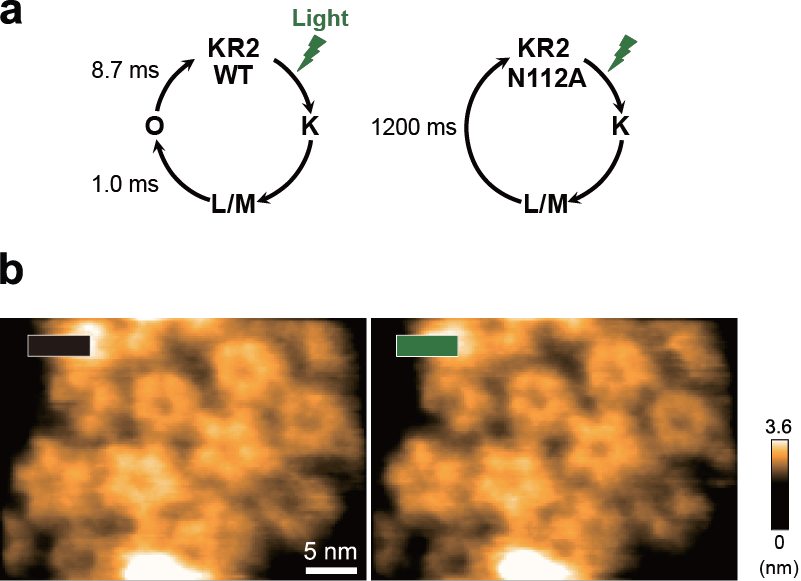
**

**Supplementary Figure S2 | HS-AFM images of KR2_278_ N112A.** (**a**) Photocycle of wild-type (left) and N112A (right) KR2. (**b**) Representative HS-AFM images of KR2_278_ N112A in the dark and under the illumination condition. A green bar indicates green light illumination. Frame rate, 2 fps.

**
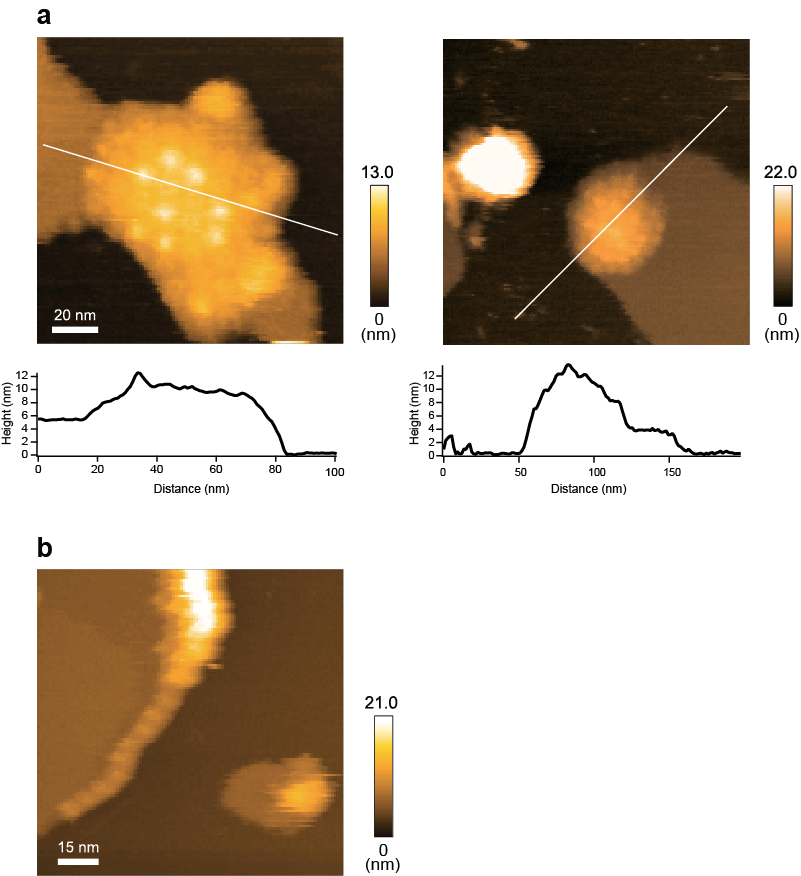
**

**Supplementary Figure S3 | Typical AFM images of KR2 reconstituted in liposomes.** The liposome was composed of POPE and POPG (molar ratio = 3:1). (**a**) AFM images and cross-sectional height profiles of ruptured proteoliposome in which the molar ratio between protein and lipids was 1:20. KR2 molecules are highly packed in the curved membrane. (**b**) AFM images obtained for ruptured proteoliposome in which the molar ratio between protein and lipids was 1:100. KR2 molecules are mainly observed at the edge of membranes.

**Supplementary Video S1 | The HS-AFM movie of KR2_278_ N112A under periodical light irradiation.** Frame rate, 2 fps.
